# Study of one-dimensional nonlinear lattice

**Published:** 2004-10-01

**Authors:** Morikazu Toda

**Affiliations:** Emeritus Professor, Tokyo University of Education

**Keywords:** Dual system, nonlinear lattice, Toda lattice, Yang-Yang’s thermodynamic formalism

## Abstract

In this article a brief review of the theory of one-dimensional nonlinear lattice is presented. Special attension is paid for the lattice of particles with exponential interaction between nearest neighbors (the Toda lattice). The historical exposition of findings of the model system, basic equations of motion, special solutions, and the general method of solutions are given as chronologically as possible. Some reference to the Korteweg-de Vries equation is also given. The article consists of three parts. Firstly, the idea of dual system is presented. It is shown that the roles of masses and springs of a harmonic linear chain can be exchanged under certain condition without changing the eigenfrequencies. Secondly, the idea is applied to the anharmonic lattice and an integrable lattice with exponential interaction force between adjacent particles is obtained. Special solutions to the equations of motion and general method of solution are shown. In the last part, some studies on the Yang-Yang’s thermodynamic formalism is given.

## Introduction

It was around 1960 when we organized a group of physicists especially interested in the results of computer experiments just becoming available.1) It was shown that some perturbation methods rather frequently used in physics failed in leading to correct results obtained by numerical computations. For example, the vibrational states of a lattice studied by computer exhibited sharp localization around light-mass impurities, whereas perturbation method predicted only small spreading of the frequency spectrum.2)–4) As another example, enhancement of heat flow due to nonlinearlity of the interaction force between crystal atoms was shown by the computer, whereas common theories based on approximate method predicted the opposite effect.5)

## Dual lattice

I first examined the eigenfrequencies of the simplest systems, which were one-dimensional lattice with linear interaction between particles. I examined lattices which contain mass impurities (different masses) with those containing interaction impurities (different force constants), to find that different lattices of the different sort may have the same frequency spectra. I considered the reason, and found the following mechanical theorem.6)

Consider a linear lattice of *N* particles with masses *m*_1_*, m*_2_, …, *m_N_*. If the force constants of the springs are *K*_1_*, K*_2_, …, *K_N_*, the total Hamiltonian is

[1] $$H\;\left(p,x\right)=\sum_{j=1}^{N}\frac{p_{j}^{2}}{2m_{j}}+\sum_{j=1}^{N}\frac{K_{j}}{2}{\left(x_{j}-x_{j-1}\right)}^{2}$$

where *x_j_* and *p_j_* are canonical coordinate and momentum. We put *x*_0_ = 0, *x_j_* = *r*_1_ + *r*_2_ + ····+ *r_j_*. Then the relative coordinate *r_j_* = *x_j_* − *x_j_*_−1_ can be used as the generalized coordinate, together with the conjugate momentum *s_j_* = *∂H/∂ṙ_j_*. We see

[2] $$s_{j}-s_{j+1}=p_{j}.$$

Further we may exchange the roles of the generalized coordinates and momenta writing

[3] $$r_{j}=P_{j}/a,\;\;\;s_{j}=-aQ_{j}$$

here *a* is an arbitrary constant. Then the Hamiltonian is transformed to

[4] $$\begin{array}{c} H\;\left(P,Q\right)=\sum_{j=1}^{N}\frac{1}{2m_{j}^{*}}P_{j}^{2}+\sum_{j=1}^{N}\frac{K_{j}^{*}}{2}{\left(Q_{j+1}-Q_{j}\right)}^{2},\\ Q_{N+1}=0, \end{array}$$

where

[5] $$\frac{1}{m_{j}^{*}}=\frac{K_{j}}{a^{2}},\;\;\;K_{j}^{*}=\frac{a^{2}}{m_{j}}.$$

The lattice thus obtained consists of particles with the masses $m_{1}^{*},m_{2}^{*},\ldots$ and the spring constants $K_{1}^{*},K_{2}^{*},\ldots$. Lattices expressed by *H*(*p,x*) and *H*(*P,Q*) have the same spectra, and may be called dual to each other.

## Nonlinear lattice

The problem of wave motion in nonlinear media is interesting not only as a purely mechanical problem, but also in connection with many physical phenomena such as shallow water waves, plasma waves, and heat conduction in crystals. Thus nonlinear phenomena have infinite variety compared with linear cases. It seems quite important to find feasible mathematical models to extend our way of maneuvering nonlinearity.

Vibration of a system of particles joined by harmonic springs can be described by superposition of normal modes which are mutually independent. If we excite a normal mode, its energy is not transfered to other normal modes. The system of harmonic oscillators never reaches the state of thermal equilibrium, and is non-ergodic. Since the presence of nonlinear terms may make the calculation insurmountably complex, usually it is assumed that the nonlinear terms guarantee the ergodicity and approach to the state thermal equilibrium.

Fermi, Pasta, and Ulam (FPU) intended to verify this expectation by computer experiments.7) Unfortunately, because of the ill conditions after the world war II, I could not see FPU’s paper at that time, because it was printed only as a report of the Los Alamos research center. However I had some information about FPU through the works by J. Ford and some others.8),9) It was that, contrary to the expectation of FPU, it was clarified that one-dimensional nonlinear lattices marvellously sustained the character of linear lattices.

I happened to believe as follows: There will be some model system, expressible in terms of simple mathematical formula, and admits truely exact analytic solutions. I started to seek out the soluble model, and actually found it before 1967.10)–15)

The equations of motion of a uniform one-dimensional lattice are usually written in the form

[6] $$m{\ddot{x}}_{n}=-V^{\prime }(x_{n}-x_{n-1})+V^{\prime }(x_{n+1}-x_{n})$$

where *V* (*r*) stands for the interaction potential between adjacent particles. I thought it could be more favorable to use the transformation described in the preceding section, which exchanges the role of the interaction terms with that of the momentum. Then the Hamiltonian for the above equations of motion turns to be

[7] $$H=\sum_{n=0}^{N}V(r_{n})+\frac{1}{2m}\sum_{n=0}^{N}{\left(s_{n}-s_{n-1}\right)}^{2}.$$

The canonical equations of motion are then given as

[8] $$\begin{array}{l} {\dot{r}}_{n}=\frac{\partial H}{\partial s_{n}}=\frac{1}{m}(2s_{n}-s_{n-1}-s_{n+1}),\\ {\dot{s}}_{n}=-\frac{\partial H}{\partial r_{n}}=-\frac{dV(r_{n})}{dr_{n}}. \end{array}$$

We confine ourselves to the case where the last equation is solved for *r_n_* in such a way that

[9] $$r_{n}=-\frac{1}{m}\chi ({\dot{s}}_{n}).$$

Then we have the fundamental equation

[10] $$\frac{d}{dt}\chi ({\dot{s}}_{n})=-2s_{n}+s_{n-1}+s_{n+1}.$$

Sometimes it is convenient to use

[11] $$S_{n}=\int_{\;}^{t}s_{n}\;dt$$

to write the above equation in the form

[12] $$\chi ({\ddot{S}}_{n})=-2S_{n}+S_{n-1}+S_{n+1}.$$

## Finding of the integrable lattice

We have to find the potential function *V* (*r*), or its inverse function *χ*(*⋅_n_*), which makes the above equations of motion satisfied for some non-trivial solutions. It is also hoped that the form of *V* (*r*) has some similarity with that of the intermolecular potential.

The above equations of motion looks like a recurrence formula for a periodic function by which *s_n_*_+1_ is derived from *s_n_*_−1_ and *s_n_*. In the linear case *s_n_* can be trigonometrical (sinusoidal) functions. Then, for nonlinear lattice, how about elliptic functions? I tried for some days in vain. Then an idea came about, and I tried the square sn^2^(*u*) of the Jacobian elliptic function sn(*u*). I found the addition formula for the Jacobian elliptic function

[13] $${\text{sn}}^{2}\;(u+v)-{\text{sn}}^{2}\;(u-v)=2\frac{d}{dv}\frac{\text{sn }u\;\text{cn }u\;\text{dn }u\;{\text{sn}}^{2}\;v}{1-k^{2}\;{\text{sn}}^{2}\;u\;{\text{sn}}^{2}\;v}$$

where all of the elliptic functions sn, cn, dn are of the same modulus *k*, and by definition

[14] $${\text{cn}}^{2}\;u=1-{\text{sn}}^{2}\;u,\;\;\;{\text{dn}}^{2}\;u=1-k^{2}\;{\text{sn}}^{2}\;u.$$

We define a function *ɛ*(*u*) by

[15] $$ɛ\;\left(u\right)=\int_{0}^{u}{\text{dn}}^{2}\;u\;du$$

and use the formula

[16] $$\begin{array}{c} \frac{d}{du}\;\text{sn }u=\text{cn }u\;\text{dn }u,\;\;\;\frac{d}{du}\;\text{cn }u=-\text{sn }u\;\text{dn }u,\\ \frac{d}{du}\;\text{dn }u=-k^{2}\;\text{sn }u\;\text{cn }u, \end{array}$$

to have

[17] $$\begin{array}{l} {ɛ}^{\prime }\left(u\right)={\text{dn}}^{2}\;u,\\ {ɛ}^{\prime \prime }\left(u\right)=-2k^{2}\;\text{sn }u\;\text{cn }u\;\text{dn }u. \end{array}$$

Integrating [13] with respect to *v*, we get

[18] $$ɛ\;\left(u+v\right)+ɛ\;\left(u-v\right)-2ɛ\;\left(u\right)=\frac{{ɛ}^{\prime \prime }\left(u\right)}{1/{\text{sn}}^{2}\;v-1+{ɛ}^{\prime }(u)}.$$

Though *ɛ*(*u*) is not a periodic function, Jacobian zn function and the derivative defined by

[19] $$Z\;\left(u\right)=ɛ\;\left(u\right)-\frac{E}{K}u,\;\;\;Z^{\prime }\left(u\right)={\text{dn}}^{2}\;u-\frac{E}{K}$$

are periodic functions with the period 2*K*, where *K* and *E* are respectively the complete elliptic integrals of the first and the second kind. Using these functions we rewrite the addition formula as

[20] $$Z(u+v)+Z(u-v)-2Z(u)=\frac{d}{du}\;\text{log }\left[1+\frac{Z^{\prime }(u)}{1/{\text{sn}}^{2}\;v-1+E/K}\right].$$

This is to be compared with [10].

Then we find that we have obtained a periodic wave given as

[21] $$s_{n}=\frac{2K\nu }{b/m}Z\;\left(u\right)$$

where *b* is a constant, and

[22] $$u=2\;\left(\nu t\pm \frac{n}{\lambda }\right)\;K,\;\;\;v=2K/\lambda .$$

Here *ν* (the frequency) and *λ* (the wavelength) are constants, and since *du* = 2*Kνdt*, by comparing [10] with [20] and [21], we get

[23] $$\chi (\dot{s})=\frac{m}{b}\;\text{log }\left[1+\frac{\left(b/m\right)/{\left(2K\nu \right)}^{2}}{1/{\text{sn}}^{2}\;v-1+E/K}\dot{s}\right]-m\sigma$$

where *b* and *σ* are constants. *χ*(*⋅*) = −*mr* is the inverse function of *⋅* = −*V*′(*r*), which must not contain *ν* and *v*. It means that the factor

[24] $$\frac{(b/m)/{\left(2K\nu \right)}^{2}}{\left(1/{\text{sn}}^{2}\;(2K/\lambda ))-1+(E/K\right)}=\frac{1}{a}$$

is a constant. Refering to [23] we find that

[25] $$\chi (\dot{s})=\frac{m}{b}\;\text{log }\left(1+\frac{\dot{s}}{a}\right)-m\sigma$$

and further by [9], that

[26] $$r=-\frac{1}{b}\;\text{log }\left(1+\frac{\dot{s}}{a}\right)+\sigma$$

or, solving for *⋅*, and referring to [8] we obtain

[27] $$V^{\prime }(r)=-a\;\left(e^{-b(r-\sigma )}-1\right).$$

Integrating, we finally obtain

[28] $$V(r)=\frac{a}{b}e^{-b(r-\sigma )}+ar+\text{const.}$$

In what follows, we use simpler expression by putting *σ* = 0 to write

[29] $$V(r)=\frac{a}{b}e^{-br}+ar\;\;\;(ab>0).$$

**Fig. 1:**
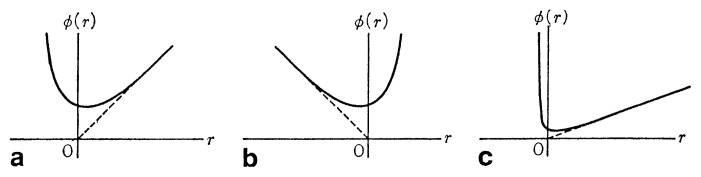
Exponential interaction potential. (a) *a, b >* 0, (b) *a, b <* 0, (c) when *b* is quite large.

This is called the Toda potential (Fig. 1). For small *r* we have

[30] $$V(r)=\frac{ab}{2}r^{2}-\frac{ab^{2}}{6}r^{3}+\cdots .$$

Thus for sufficiently small oscillation, the lattice looks like a linear lattice with the spring constant *κ* = *ab*. For somewhat large motion, the lattice will behave like a system of hard spheres.

We shall summarize the results. The equations of motion given by [6] and [29],

[31] $$m\frac{d^{2}x_{n}}{dt^{2}}=a\;\left(e^{-br_{n}}-e^{-br_{n+1}}\right),$$

is called the Toda lattice (exponential lattice) equation, which is also written as

[32] $$m\frac{d^{2}r_{n}}{dt^{2}}=a\;\left(2e^{-br_{n}}-e^{-br_{n-1}}-e^{-br_{n+1}}\right).$$

In terms of the dual lattice, by [26]

[33] $$r_{n}=-\frac{1}{b}\;\text{log }\left(1+\frac{{\dot{s}}_{n}}{a}\right)$$

we have the equations of motion

[34] $$\frac{d}{dt}\;\text{log}(a+{\dot{s}}_{n})=\frac{b}{m}(s_{n-1}-2s_{n}+s_{n+1})$$

or

[35] $$\text{log }\left(1+\frac{{\ddot{S}}_{n}}{a}\right)=\frac{b}{m}(S_{n-1}-2S_{n}+S_{n+1})$$

where by [33] the relation between *r_n_* and *⋅_n_* is given as

[36] $${\dot{s}}_{n}=a\;\left(e^{-br_{n}}-1\right).$$

**Fig. 2:**
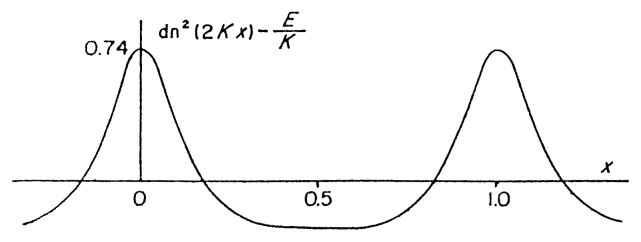
Cnoidal wave, dn^2^(2*Kx*)−*E/K* as a function of *x*, for *k*^2^ = 0*.*992 when *k* is the modulus.

The periodic wave solution is given by [21], [36] and [19] as (cnoidal wave, Fig. 2)

[37] $$e^{-br_{n}}-1=\frac{{\left(2K\nu \right)}^{2}}{ab/m}\;\left\{{\text{dn}}^{2}2\;\left(\frac{n}{\lambda }\pm \nu t\right)\;K-\frac{E}{K}\right\}$$

with the dispersion relation which is given by [24] as

[38] $$2K\nu =\sqrt{\frac{ab}{m}}/\sqrt{\frac{1}{{\text{sn}}^{2}\;(2K/\lambda )}-1+\frac{E}{K}}$$

where *K* and *E* are complete elliptic integrals

[39] $$\begin{array}{l} K=K(k)=\int_{0}^{\pi /2}\frac{d\theta }{\sqrt{1-k^{2}\;{\text{sin}}^{2}\;\theta }},\\ E=E(k)=\int_{0}^{\pi /2}\sqrt{1-k^{2}\;{\text{sin}}^{2}\;\theta }d\theta . \end{array}$$

Thus I found the nonlinear lattice and its periodic solutions at the same time.

## Continuous limit

Sometimes it is seen that the continuum approximation gives good results. In this case it is convenient to use the operator rule

[40] $$e^{\pm d/dn}f(n)=f(n\pm 1).$$

Then we see the simplified version *a* = *b* = *m* = 1 of the equations of motion [32] can be written as

[41] $$\frac{\partial^{2}\;r}{\partial t^{2}}+{\left[2\;\text{sinh }\left(\frac{1}{2}\frac{\partial }{\partial n}\right)\right]}^{2}\;e^{-r}=0.$$

If we straightly expand the left hand side in powers of *∂/∂n* and neglect higher powers of *r*, we can rewrite the above equation as

[42] $$\left(\frac{\partial }{\partial t}-2\;\text{sinh}\;\frac{1}{2}\frac{\partial }{\partial n}+\frac{1}{2}\frac{\partial }{\partial n}r\right)\times \left(\frac{\partial }{\partial t}+2\;\text{sinh}\;\frac{1}{2}\frac{\partial }{\partial n}-\frac{1}{2}r\frac{\partial }{\partial n}\right)\;r=0.$$

Thus, for the wave advancing to the right, we have

[43] $$\left(\frac{\partial }{\partial t}+2\;\text{sinh}\;\frac{1}{2}\frac{\partial }{\partial n}-\frac{1}{2}r\frac{\partial }{\partial n}\right)\;r=0$$

and further expanding sinh,

[44] $$\left(\frac{\partial }{\partial t}+\frac{\partial }{\partial n}-\frac{1}{12}\frac{\partial^{3}}{\partial n^{3}}-\frac{1}{2}r\frac{\partial }{\partial n}\right)\;r=0.$$

If we change the units and signs, we can arrive at an equation of the form

[45] $$\frac{\partial u}{\partial t}+u\frac{\partial u}{\partial x}+\delta^{2}\frac{\partial^{3}\;u}{\partial x^{3}}=0.$$

**Fig. 3:**
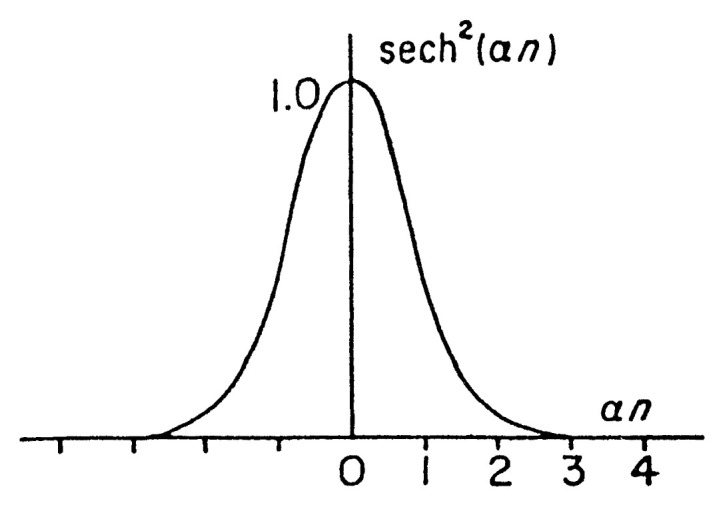
Soliton, sech^2^(*αn*) as a function of *αn*.

This is the famous Korteweg-de Vries (KdV) equation originally derived to describe the shallow water waves.16) They gave periodic solution which is similar to the periodic wave given in the foregoing solution. Besides they gave solution of the form (Fig. 3)

[46] $$u=u_{\infty }+A\;{\text{sech}}^{2}\;(\alpha x-\beta t)$$

which is a solitary wave solution or soliton solution.

N. Zabusky and M. D. Kruskal17) studied the KdV equation by computer experiment. They used the cyclic boundary conditions and started from the initial state such as

[47] u(t=0)=A cos πx.

They found the wave split into a group of solitary waves (solitons), each of which proceeded nearly independently and after a while the wave recovered the initial state.

If the wave consists of a small number of solitons it will come back to the initial state after a time, which is nearly equal to the least common multiple of the recurrence times of the solitons.

## Solitons

A soliton of the nonlinear lattice can be thought as the limit of infinite wave length. It leads to a soliton solution[Fig f4a-pjab-80-445][Fig f4b-pjab-80-445]

$$e^{-br_{n}}-1=\frac{m}{ab}\beta^{2}\;{\text{sech}}^{2}\;(\kappa n\pm \beta t)$$

with

[48] $$\beta =\sqrt{\frac{ab}{m}}\;\text{sinh}\;\kappa .$$

The velocity of the soliton is

[49] $$c=\sqrt{\frac{ab}{m}}\;\frac{\text{sinh}\;\kappa }{\kappa }$$

which is larger for the soliton of larger height.

In a paper P. D. Lax wrote two-soliton state of the KdV equation without any proof. But I found a hint from it to rewrite [48] in the form12),18)

[50] $$e^{-br_{n}}-1=\frac{m}{ab}\frac{d^{2}}{dt^{2}}\;\text{log cosh}(\kappa n-\beta t)$$

with *β* determined by the equations of motion as

[51] $$\beta =\sqrt{\frac{ab}{m}}\;\text{sinh}\;\kappa .$$

Then I found that two soliton state is given by

$$e^{-br_{n}}-1=\frac{m}{ab}\frac{d^{2}}{dt^{2}}\;\text{log }\psi_{n}$$

with

[52] $$\psi_{n}=A\;\text{cosh}(\kappa n-\beta t)+B\;\text{cosh}(\mu n-\gamma t)$$

where *A*, *B*, *β* and *γ* are determined from the equations of motion.

There are two cases. One is the head-on collision of two solutions, given by

[53] $$\begin{array}{c} \beta =\sqrt{\frac{ab}{m}}2\;\text{sinh}\;\frac{\mu }{2}\;\text{cosh}\;\frac{\kappa }{2},\;\;\;\gamma =\sqrt{\frac{ab}{m}}2\;\text{sinh}\;\frac{\kappa }{2}\;\text{cosh}\;\frac{\mu }{2}\\ B/A=\text{cosh}\;\frac{\kappa }{2}/\text{cosh}\;\frac{\mu }{2}. \end{array}$$

The other case is for two solitons running in the same direction:

[54] $$\begin{array}{c} \beta =\sqrt{\frac{ab}{m}}2\;\text{sinh}\;\frac{\kappa }{2}\;\text{cosh}\;\frac{\mu }{2},\;\;\;\gamma =\sqrt{\frac{ab}{m}}2\;\text{sinh}\;\frac{\mu }{2}\;\text{cosh}\;\frac{\kappa }{2}\\ B/A=\text{sinh}\;\frac{\kappa }{2}/\text{sinh}\;\frac{\mu }{2}. \end{array}$$

## Integrability

**Fig. 5:**
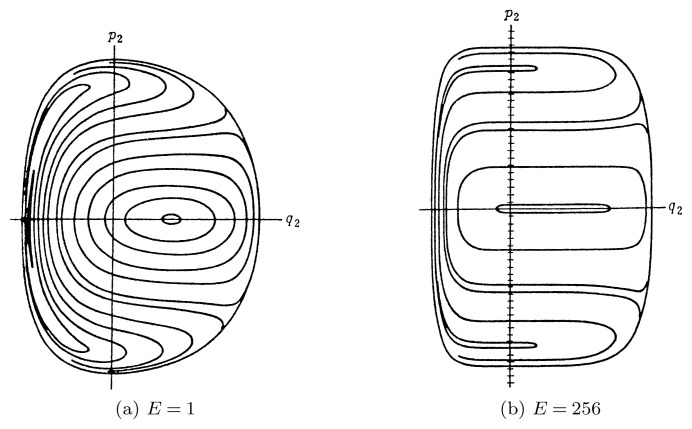
Surface of section for the Hamiltonian [55]. Poincaré mapping, Integrable case.

Ford19) numerically examined the integrability of the lattice with exponential interaction between particles. He took cyclic lattice of three particles, and applied the Poincaré method of mapping (surface of section) of trajectories in the phase space. He found that the trajectories are smooth and did not become erratic even if the energy is raised extremely high, indicating that the lattice was integrable (Fig. 5).

The Hamiltonian of a three-particle cyclic lattice with exponential interaction can be written in a dimensionless form as

[55] $$H=\frac{1}{2}(P_{1}^{2}+P_{2}^{2}+P_{3}^{2})+e^{-(Q_{1}-Q_{2})}+e^{-(Q_{2}-Q_{3})}+e^{-(Q_{3}-Q_{1})}-3.$$

He applied the transformation

$$Q_{i}=\sum_{j=1}^{3}A_{ij}\zeta_{j},\;\;\;P_{i}=\sum_{j=1}^{3}A_{ij}\eta_{j}$$

with

[56] $$A=\left(\begin{array}{ccc} 6^{-1/2} & 2^{-1/2} & 3^{-1/2}\\ -{\left(2/3\right)}^{1/2} & 0 & 3^{-1/2}\\ 6^{-1/2} & -2^{-1/2} & 3^{-1/2} \end{array}\right)$$

which diagonalizes the corresponding harmonic lattice. Then, by rescaling as

[57] $$\zeta_{1}=2\sqrt{2}q_{1},\;\;\;\zeta_{2}=2\sqrt{2}q_{2},\;\;\;t\to t/\sqrt{3}$$

he obtained the equations of motion

[58] $$\begin{array}{c} {\ddot{q}}_{1}={\left(4\sqrt{3}\right)}^{-1}\left(-e^{2q_{2}+2\sqrt{3}q_{1}}+e^{2q_{2}-2\sqrt{3}q_{1}}\right)\\ {\ddot{q}}_{2}=\frac{1}{6}e^{-4q_{2}}-\frac{1}{12}\left(e^{2q_{2}+2\sqrt{3}q_{1}}+e^{2q_{2}-2\sqrt{3}q_{1}}\right). \end{array}$$

Ford numerically integrated [58] and had the Poincaré mappings of the three-particle exponential lattice. Fig. 5(a) is for the energy *E*=1, and Fig. 5(b) is for *E*=256. He examined mapping up to *E*=56000, and always he had smooth curves with no indication of stochastic behaviour. Thus the numerical works strongly suggested that the lattice with exponential interaction is integrable. In other words, it admits the so-called third integral besides momentum and energy.

These results were in strong contrast to the behaviour of usual nonlinear system, which are in almost all cases chaotic, non-integrable.

For instance since 1964, we had the so-called Hénon-Heiles system,20) which is equivalent to a cyclic lattice of three particles with the Hamiltonian

[59] $$\begin{array}{ll} H=\; & \frac{1}{2}(P_{1}^{2}+P_{2}^{2}+P_{3}^{2})\\ & +\frac{1}{2}\;[{\left(Q_{1}-Q_{2}\right)}^{2}+{\left(Q_{2}-Q_{3}\right)}^{2}+{\left(Q_{3}-Q_{1}\right)}^{2}]\\ & +\frac{1}{6}\;[{\left(Q_{1}-Q_{2}\right)}^{3}+{\left(Q_{2}-Q_{3}\right)}^{3}+{\left(Q_{3}-Q_{1}\right)}^{3}]. \end{array}$$

The second term on the right hand side is the harmonic interaction and the third is the cubic nonlinear term. After performing the transformations [56] and [57], we obtain the equations of motion

[60] $$\begin{array}{l} {\ddot{q}}_{1}=-q_{1}-2q_{1}q_{2}\\ {\ddot{q}}_{2}=-q_{2}-q_{1}^{2}+q_{2}^{2}. \end{array}$$

As easily seen,18) if we expand the right hand side of [58] up to the second powers of *q*_1_ and *q*_2_, we get [60].

**Fig. 6:**
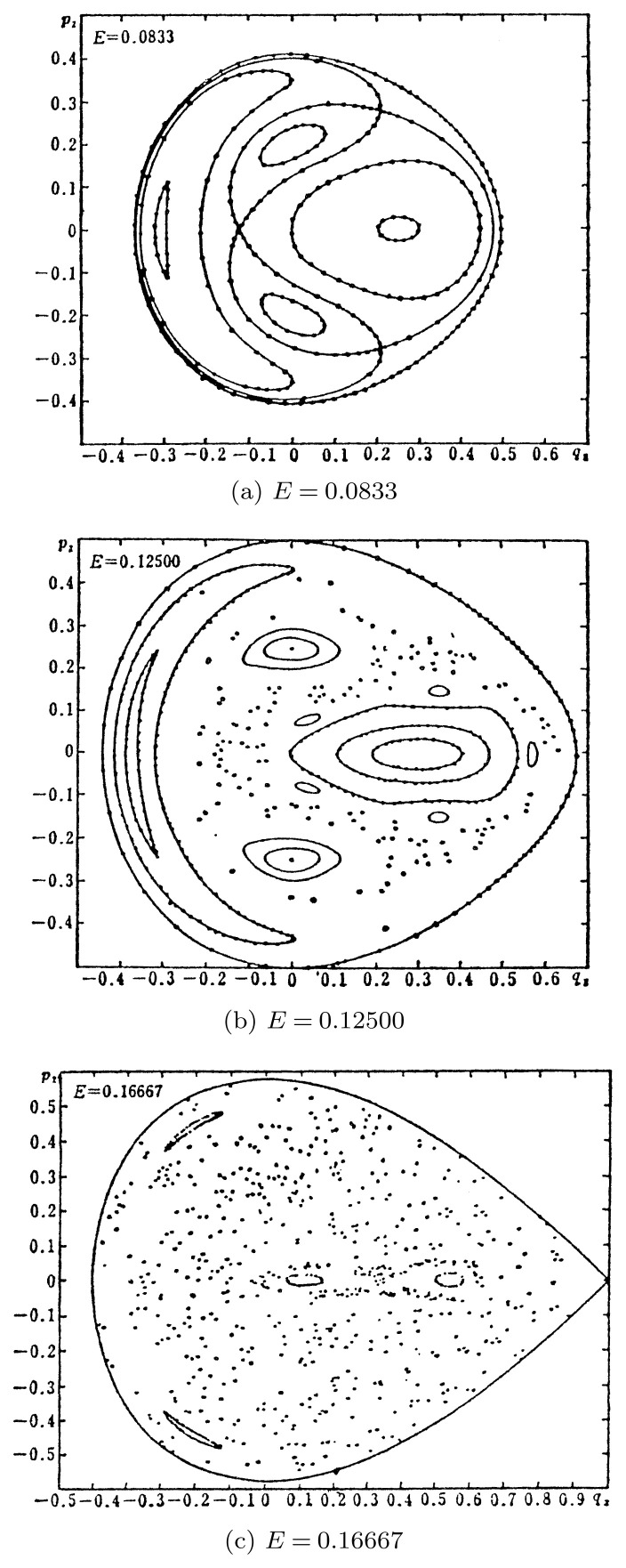
Surface of section for the Hamiltonian [59]. Poincaré mapping, Non-integrable case.

But in both cases the trajectories are quite different. In the case of [55] they are entirely smooth showing integrability of the exponential lattice. On the contrary, the trajectories of [60] become chaotic as shown in Fig. 6(a), (b) and (c) with increasing energy *E*, indicating non-integrability of the lattice with cubic nonlinearlity.

This is a very clear example of the transition between integrable and non-integrable systems.

## Conserved quantities

The equations of motion of the cyclic three-particle exponential lattice [55] can be written as

[61] $$\begin{array}{c} {\dot{P}}_{1}=X_{3}-X_{1},\;\;\;{\dot{P}}_{2}=X_{1}-X_{2},\;\;\;{\dot{P}}_{3}=X_{2}-X_{3}\\ {\dot{X}}_{1}=(P_{1}-P_{2})X_{1},\;\;\;{\dot{X}}_{2}=(P_{2}-P_{3})X_{2},\\ {\dot{X}}_{3}=(P_{3}-P_{1})X_{3}, \end{array}$$

where

[62] $$\begin{array}{c} X_{1}=e^{-(Q_{2}-Q_{1})},\;\;\;X_{2}=e^{-(Q_{3}-Q_{2})},\\ X_{3}=e^{-(Q_{1}-Q_{3})}. \end{array}$$

From these equations we see that

[63] $$\begin{array}{l} I_{1}=P_{1}+P_{2}+P_{3}\\ I_{2}=P_{1}P_{2}+P_{2}P_{3}+P_{3}P_{1}-X_{1}-X_{2}-X_{3}\\ I_{3}=P_{1}P_{2}P_{3}-P_{1}X_{2}-P_{2}X_{3}-P_{3}X_{1} \end{array}$$

are conserved quantities. *I*_1_ is the total momentum, *I*_2_ is related to the total energy ($E=(1/2)I_{1}^{2}-I_{2}$). *I*_3_ is the so-called third integral of motion, which is not interpreted in terms of momentum and energy. The existence of the third integral in this case was proved by the numerical study of the preceding section.

M. Hénon21) and H. Flaschka22) were stimulated by the results of the numerical study performed by Ford.19) Hénon proved that uniform cyclic exponential lattice has as many conserved quantities as the number of particles in the lattice. If we write them for the case of three particles, we get *I*_1_, *I*_2_ and *I*_3_ as mentioned above.

In the same year, Flaschka independently proved the same thing by a method, which we shall explain in the next section.22)

## Matrix equation of motion

We continue dealing with cyclic exponential lattice of *N* particles. We first rewrite the equations of motion23)

[64] $$\frac{d^{2}Q_{n}}{dt^{2}}=e^{-(Q_{n}-Q_{n-1})}-e^{-(Q_{n+1}-Q_{n})}$$

as

[65] $$\begin{array}{l} \frac{d}{dt}e^{-(Q_{n+1}-Q_{n})}=-(P_{n+1}-P_{n})e^{-(Q_{n+1}-Q_{n})}\\ \frac{d}{dt}P_{n}=e^{-(Q_{n}-Q_{n-1})}-e^{-(Q_{n+1}-Q_{n})}. \end{array}$$

If we put

[66] $$a_{n}=\frac{1}{2}e^{-(Q_{n+1}-Q_{n})/2},\;\;\;b_{n}=\frac{1}{2}P_{n}$$

we have

[67] $${\dot{a}}_{n}=a_{n}(b_{n}-b_{n+1}),\;\;\;{\dot{b}}_{n}=2(a_{n-1}^{2}-a_{n}^{2}).$$

For a cyclic lattice of *N* particles forming a ring

[68] $$a_{0}=a_{N},\;\;\;b_{0}=b_{N}$$

the equations of motion can be written in the matrix form as

[69] $$\frac{dL}{dt}=BL-LB.$$

This is called the Lax formalism.24) It was first introduced for the KdV equation, and afterwards used in many cases. It is assumed that *B* is anti-symmetric. In our case we have

[70] L=[b1a10⋯0aNa1b2a2 000a2b3  0⋮  ⋱ ⋮0   bN-1aN-1aN0 ⋯aN-1bN],B=[0-a10⋯0aNa10-a2 000a20  0⋮  ⋱ ⋮0   0-aN-1-aN0 ⋯aN-10].

Matrix components *a_n_* and *b_n_* are functions of the displacements and momenta, which are functions of time. Therefore matrices *L* and *B* are functions of time. We write them as *L*(*t*) and *B*(*t*), and the initial values as *L*(0) and *B*(0). We also write eigenvalue of *L*(*t*) as *λ*(*t*) to express the *t* dependence, though we will find that *λ* is independent of *t*. If we write the eigenfunction as *ϕ*(*t*) (*N* ×1 matrix), we have

[71] L(t)ϕ(t)=λ(t)ϕ(t)

where *L*(*t*) is a *N* ×*N* matrix. *λ*(*t*) are given as the roots of the equation

[72] det[L(t)-λ(t)I]=0

where *I* is the *N* ×*N* unit matrix.

Since *B* is antisymmetric the matrix *U* defined by

[73] $$\frac{dU}{dt}=BU,\;\;\;U(0)=1$$

is unitary. That is we have

[74] $$\begin{array}{c} \frac{dU^{-1}}{dt}=-U^{-1}B,\\ UU^{-1}=U^{-1}U=I. \end{array}$$

Further, by [69], we have

[75] $$\frac{d}{dt}(U^{-1}LU)=-U^{-1}\;\left[BL-\frac{dL}{dt}-LB\right]\;U=0$$

so that

[76] $$L(t)=U(t)L(0)U^{-1}(t).$$

Thus *L*(*t*) and *L*(0) are unitary equivalent.

Now, in general, if *A* and *B* are square matrices, we have

[77] det(AB)=det A det B.

That is, the determinant of a product of matrices is equal to the product of their determinants. By [69] and [77], we have

[78] $$\begin{array}{l} 0=\text{det}[L(t)-\lambda (t)I]\\ =\text{det}\{U(t)[L(0)-\lambda (t)]U^{-1}(t)\}\\ =\text{det}[L(0)-\lambda (t)I]. \end{array}$$

Comparing this result with det[*L*(0)−*λ*(0)*I*] = 0 we get

[79] $$\lambda (t)=\lambda (0),\;\;\;\frac{d\lambda }{dt}=0.$$

Therefore the eigenvalues are independent of time.

Thus it is shown that the motion in the lattice conserves its spectrum (isospectral deformation). Expanding [72], we get the eigenvalue equation

[80] $$\lambda_{j}^{N}+c_{1}\lambda_{j}^{N-1}+c_{2}\lambda_{j}^{N-2}+\cdots +c_{N-1}\lambda_{j}+c_{N}=0,\;\;\;(j=1,2,\ldots ,N)$$

where *c_k_* are functions of *a_n_* and *b_n_*. From the above simultaneous equations we can write *c_k_* as functions of *λ_j_*, which are time-independent. Therefore *c_k_* are also conserved quantity. It is verified that *c_k_* are essentially the same as Hénon’s constants of motion. We may also obtain these constants of motion as

[81] $$J_{p}=\text{trace }L^{p}=\sum_{j=1}^{N}\lambda_{j}^{p},\;\;\;(p=1,2,\ldots ,N).$$

Thus it is shown that cyclic exponential lattice of *N* particles has the same number of conserved quantities, which means that it is integrable.

## Inverse scattering method

If we write down the eigenvalue equation [71], we have

[82] $$a_{n-1}\phi_{n-1}+b_{n}\phi_{n}+a_{n}\phi_{n+1}=\lambda \phi_{n}.$$

This equation has its counter-part in the case of the KdV equation.

Consider a soliton with negative value

[83] $$u(x,t)=-\kappa^{2}\;{\text{sech}}^{2}\;(\kappa x-4\kappa^{3}\;t+\delta ).$$

This is an example of solutions of the KdV equation

[84] $$u_{t}-6uu_{x}+u_{xxx}=0.$$

Suppose we have a wave under appropriate boundary conditions and develops with time according to the above KdV equation. Then the eigenvalues *λ* of the Schrödinger type equation

[85] $$\left(-\frac{\partial^{2}}{\partial x^{2}}+u\right)\;\psi =\lambda \psi$$

stay independent of time, so that

[86] $$\frac{d\lambda }{dt}=0.$$

Equations [85] and [86] are the KdV version of the discrete case [71] and [79].

Starting from this finding, Gardner, Greene, Kruskal and Miura invented a method of solution to the KdV equation,25) which solves the initial value problem and called as the inverse scattering method.

For the case of discrete lattice, H. Flaschka23) applied the inverse scattering method to the Toda lattice. The motion in the infinite Toda lattice was also solved by E. Date and S. Tanaka.26) Using the theory of hyper elliptic integrals M. Kac and P. van Moerbeke showed similar method to solve the three particle cyclic lattice.27)

## Probability distribution

We now turn to another problem. Around 1940, that is when I graduated from the university, there were senior friends in Japan studying thermodynamical problems such as the distribution function of molecules in liquids and possibility of phase transition to gases or solid state, and so on. T. Nagamiya28) considered one dimensional chain of particles where the total potential is the sum of the potential *V* (*x_n_* − *x_n_*_−1_) between adjacent particles. The distribution *g_n_*(*x*) of the *n*-th particle is governed by

[87] $$g_{n}(x)=\int_{-\infty }^{\infty }g(x-x^{\prime })g_{n-1}(x^{\prime })dx^{\prime }$$

with

[88] $$g(x)=\frac{e^{-\beta V(x)-\beta fx}}{\int_{-\infty }^{\infty }e^{-\beta V(x^{\prime })-\beta fx^{\prime }}dx^{\prime }}$$

for the canonical ensemble (*f* = pressure).

Nagamiya calculated the distribution function *g_n_*(*x*) for some special potentials, such as the harmonic potential and hard sphere potential. The results were to be compared with the distribution function of molecules revealed by the X-ray experiments of these days.

## Partition function

H. Takahashi29) invented a simple method for treating the statistical mechanics of one dimensional chain of *N* molecules. He considered the configurational integral of the system (*β* = 1*/kT*)

[89] $$Q_{N}(x_{n})=\int_{x_{0}<x_{1}}\cdots \int_{<\cdots <x_{N}}dx_{1}\;dx_{2}\cdots dx_{N-1}\times e^{-\beta \{V(x_{1})+V(x_{2}-x_{1})+\cdots +V(x_{N}-x_{N-1})\}}.$$

Instead of the variables *x*_1_*, x*_2_, …, *x_N_*, we introduce

[90] $$r_{1}=x_{1},\;r_{2}=x_{2}-x_{1},\;\ldots ,\;r_{N}=x_{N}-x_{N-1}$$

and observe the Jacobian

[91] $$\frac{\partial (r_{1},r_{2},\ldots ,r_{N})}{\partial (x_{1},x_{2},\ldots ,x_{N})}=1.$$

Then we see that

[92] $$\int_{0}^{\infty }Q_{N}(x_{N})e^{-\beta fx_{N}}\;dx_{N}={\left[Q(\beta )\right]}^{N}$$

where we have written *Q*(*β*) for

[93] $$Q(\beta )=\int_{0}^{\infty }\text{exp }[-\beta \{V(r)+fr\}]\;dr$$

which is the configurational part of the partition function. By the standard statistical mechanical argument we see that the average length of the system is given as

[94] $$l=\frac{{\bar{x}}_{N}}{N}=-\frac{\partial }{\partial f}kT\;\text{log }Q(\beta )$$

and the energy per particle by

[95] $$E=\frac{1}{2}kT-\frac{\partial }{\partial \beta }\;\text{log }Q(\beta ).$$

We see that the length *l* is always a single valued function of the pressure, which means that no phase change occurs in one dimensional system.

In classical mechanics, the Hamiltonian per particle is

[96] $$H(p,x)=\frac{1}{2}p^{2}+V(x)+fx.$$

If we write the total partition function as *ζ*(*β*), it is given by

[97] $$\begin{array}{l} \zeta (\beta )=\int_{-\infty }^{\infty }dp\int_{-\infty }^{\infty }dq\;\text{exp }[-\beta H(p,x)]\\ =\sqrt{\frac{2\pi }{\beta }}Q(\beta ). \end{array}$$

## Exponential lattice

In the case of the exponential lattice, we can use the simple expression

[98] $$V(x)=e^{-x}.$$

We have

[99] $$Q(\beta )=\int_{-\infty }^{\infty }\text{exp }\left[-\beta \;\left(e^{-x}+fx\right)\right]\;dx.$$

To perform the integration we put *e*^−^*^x^* = *t*, and get

[100] $$\begin{array}{l} Q(\beta )=\int_{0}^{\infty }e^{-\beta t}t^{\beta f-1}dt=\beta^{-\beta f}\int_{0}^{\infty }e^{-y}y^{\beta f-1}dy\\ =\beta^{-\rho }\Gamma (\rho ) \end{array}$$

where Γ(*ρ*) is the gamma function of the order *ρ* = *βf.* [101]

[101] ρ=βf.

## Thermodynamics

The Bethe anzatz was first invented for quantum-mechanical one-dimensional spin system, and extended to one-dimensional system of particle interacting via a repulsive delta-function potential. It was further developed by C. N. Yang and C. P. Yang to the thermodynamical system with given temperature and pressure.30)

Following N. Theodorakopoulos,31) the classical limit of the Yang-Yang’s thermodynamical equation written for the chemical potential *μ* is

[102] $$\frac{1}{2}p^{2}-\mu -ɛ(p)+\frac{2}{\beta }\int_{-\infty }^{\infty }\theta^{\prime }(p-p^{\prime })e^{-\beta ɛ(p^{\prime })}dp^{\prime }=0$$

with the subsidiary condition

[103] $$\beta f=\int_{-\infty }^{\infty }e^{-\beta ɛ(p^{\prime })}dp^{\prime }$$

where *f* is used for the pressure to discriminate it from momentum. In the above integral equation the factor exp(−*βε*) is related to some kind of excitation, and *θ*′(*p*) stands for the derivative of the two-body scattering phase shift *θ*(*p*) for the interaction potential, and *p* denotes the relative momentum. In the classical limit, and for the exponential interaction, we use

[104] $$\theta^{\prime }(p)=-2\;\text{log}\;|p|.$$

then the above equation is rewritten as

[105] $$-\beta \mu =-\beta \frac{p^{2}}{2}+\beta ɛ(p)+2\int_{-\infty }^{\infty }\text{log}\;|p-p^{\prime }|e^{-\beta ɛ(p^{\prime })}dp^{\prime }.$$

By setting

[106] $$\begin{array}{c} x=\beta^{1/2}p,\;\;\;\beta^{1/2}\varphi (x)=e^{-\beta ɛ(p)},\;\;\;\rho =\beta f,\\ \psi (x)=\int_{-\infty }^{\infty }\text{log}\;\frac{|x-x^{\prime }|}{\sqrt{\beta }}\;\varphi (x^{\prime })dx^{\prime } \end{array}$$

we have

[107] $$e^{-\beta \mu }=\frac{e^{-\frac{1}{2}x^{2}}\;e^{2\psi (x)}\;}{\sqrt{\beta }\varphi (x)}$$

and

[108] $$\rho =\int_{-\infty }^{\infty }\varphi (x^{\prime })dx^{\prime }.$$

The meaning of the Yang-Yang equation [102] is as follows: Consider that the scattering function *θ*(*p*) is given by experiment. Then [102] is solved for *ε*(*p*), under the subsidiary condition [103], which determines the chemical potential *μ*.

## Solution

In order to have the solution *ϕ*(*x*), we differentiate [107] with respect to *x* and obtain

[109] $$\varphi^{\prime }(x)+x\varphi (x)=2\varphi (x)\psi^{\prime }(x).$$

This is nonlinear with respect to *ϕ*(*x*) for *ψ*(*x*) implicitly contains *ϕ*(*x*). M. Opper solved this equation.32) The result can be written as

[110] $$\text{exp }\left[-\psi (x)+i\pi \int_{0}^{x}\varphi (x^{\prime })dx^{\prime }\right]=C\int_{0}^{\infty }e^{ixt}e^{-t^{2}/2}t^{\rho -1}dt$$

where the constant *C* is determined as

[111] $$C=\beta^{\rho /2}/\Gamma (\rho )$$

comparing the asymptotic behavior of both sides of [110] for *x*→*∞*.

To show that *ϕ*(*x*) and *ψ*(*x*) given by [110] satisfy [109], we introduce real functions *R*(*x*) and *J*(*x*) by

[112] $$C\int_{0}^{\infty }e^{ixt}e^{-t^{2}/2}t^{\rho -1}dt=C\{R(x)+iJ(x)\}=C\sqrt{R{\left(x\right)}^{2}+J{\left(x\right)}^{2}}e^{i\text{arctan}(J(x)/R(x))}$$

where

[113] $$\begin{array}{l} R(x)=\int_{0}^{\infty }\text{cos }xt\;e^{-t^{2}/2}t^{\rho -1}dt,\\ J(x)=\int_{0}^{\infty }\text{sin }xt\;e^{-t^{2}/2}t^{\rho -1}dt. \end{array}$$

Comparing the both sides of [110], we get

[114] $$\begin{array}{l} \psi (x)=\text{log}\;C\sqrt{R{\left(x\right)}^{2}+J{\left(x\right)}^{2}},\\ \psi^{\prime }(x)=-\frac{RR^{\prime }+JJ^{\prime }}{R^{2}+J^{2}},\\ \varphi (x)=\frac{d}{dx}\frac{1}{\pi }\;\text{arctan}\;\frac{J(x)}{R(x)}\\ =\frac{1}{\pi }\frac{RJ^{\prime }-R^{\prime }J}{R^{2}+J^{2}}, \end{array}$$

to see

[115] $$\varphi^{\prime }(x)+x\varphi (x)=\frac{1}{\pi }\frac{\left(RJ^{\prime \prime }-R^{\prime \prime }J)+x(RJ^{\prime }-R^{\prime }J\right)}{R^{2}+J^{2}}+\varphi (x)\psi^{\prime }(x).$$

Since

[116] $$J^{\prime }=\int_{0}^{\infty }\text{cos }xt\;e^{-t^{2}/2}t^{\rho }dt$$

by integrating partially we have

[117] $$\begin{array}{l} xJ^{\prime }=\int_{0}^{\infty }\frac{d}{dt}(\text{sin }xt)e^{-t^{2}/2}t^{\rho }dt\\ ={\text{sin }xt\;e^{-t^{2}/2}t^{\rho }|}_{0}^{\infty }-\int_{0}^{\infty }\text{sin }xt\frac{d}{dt}\left(e^{-t^{2}/2}t^{\rho }\right)\;dt\\ =-\int_{0}^{\infty }\text{sin }xt\;e^{-t^{2}/2}\left(-t^{\rho +1}+\rho t^{\rho -1}\right)\;dt\\ =-\int_{0}^{\infty }\text{sin }xt\;e^{-t^{2}/2}t^{\rho +1}dt-\rho J\\ =-J^{\prime \prime }-\rho J. \end{array}$$

Similarly we have

[118] $$xR^{\prime }=-R^{\prime \prime }-\rho R.$$

So that

[119] $$RJ^{\prime \prime }-R^{\prime \prime }J+x(RJ^{\prime }-R^{\prime }J)=0$$

and [109] is satisfied.

Therefore the solution *ϕ*(*x*) and *ψ*(*x*) of the Yang-Yang equation [110] for the exponential lattice is given by [114] in terms *R*(*x*) and *J*(*x*). Then the chemical potential *μ* for each particle is given by [107].

## Chemical potential

However, since in [107] the chemical potential *μ* is independent of *x*, we may put *x* = 0 in the right hand side of this equation to estimate *μ*, so that

[120] $$e^{-\beta \mu }-\frac{e^{2\psi (0)}}{\sqrt{\beta }\varphi (0)},$$

with

[121] $$\psi (0)=\int_{-\infty }^{\infty }\text{log}(|x^{\prime }|/\sqrt{\beta })\varphi (x^{\prime })dx^{\prime }.$$

Following the standard theory of classical statistical mechanics, we see that *e*^−^*^βμ^* is nothing but the partition function *ζ*(*β*) calculated as [97] and [100]:

[122] $$\zeta (\beta )=\sqrt{\frac{2\pi }{\beta }}Q(\beta ),\;\;\;Q(\beta )=\beta^{-\rho }\Gamma (\rho ).$$

On the other hand, we have *J*(0) = *R*′(0) = 0, and from [113] and [114]

[123] $$\begin{array}{l} e^{\psi (0)}=\frac{\Gamma (\rho )}{\beta^{\rho /2}}\;{\left(\int_{0}^{\infty }e^{-t^{2}/2}t^{\rho -1}dt\right)}^{-1}\\ =\frac{\sqrt{2/\pi }}{\beta^{\rho /2}}\int_{0}^{\infty }e^{-t^{2}/2}t^{\rho }dt\\ =\frac{\beta^{-\rho /2}}{2^{\rho /2-1}}\frac{\Gamma (\rho )}{\Gamma (\rho /2)},\\ \varphi (0)=\frac{\int_{0}^{\infty }e^{-t^{2}/2}t^{\rho }dt}{\pi \int_{0}^{\infty }e^{-t^{2}/2}t^{\rho -1}dt}\\ =\frac{1}{\sqrt{2\pi }}\frac{1}{2^{\rho -2}}\frac{\Gamma (\rho )}{{\left[\Gamma (\rho /2)\right]}^{2}}. \end{array}$$

Therefore [120] gives

[124] $$e^{-\beta \mu }=\sqrt{\frac{2\pi }{\beta }}\beta^{-\rho }\Gamma (\rho ).$$

Thus we have reproduced the formula *ζ*(*β*) = *e*^−^*^βμ^*.

Now, our problem is to clarify the meaning of *ψ*(0) and *ϕ*(0) by identifying them to well-known statistical mechanical quantities such as the phase integral or some part of partition function.

## Binary interaction

In [120] the factor *e*^2^*^ψ^*^(0)^ looks indicating pairing of two factors of *ψ*(0), which seems to come from the pair of interaction potentials giving rise to

[125] $$\begin{array}{l} \frac{1}{2}(e^{-x}+fx)+\frac{1}{2}(e^{-y}+fy)=e^{-(x+y)/2}\frac{1}{2}\;\left[e^{(y-x)/2}+e^{-(y-x)/2}\right]+\frac{f}{2}(x+y)\\ =e^{-\xi }\;\text{cosh}\;\frac{\eta }{2}+f\xi \\ =e^{-[\xi -\bar{\xi }(\eta )]}+f[\xi -\bar{\xi }(\eta )]+f\bar{\xi }(\eta ) \end{array}$$

with

[126] $$\begin{array}{c} e^{\bar{\xi }(\eta )}=\text{cosh}(\eta /2),\\ \xi =\frac{x+y}{2},\;\;\;\eta =y-x. \end{array}$$

The transformation from (*x,y*) to (*ξ,η*) is a canonical transformation,

[127] $$\frac{\partial (\xi ,\eta )}{\partial (x,y)}=1,\;\;\;dx\;dy=d\xi \;d\eta .$$

Integrating we find the formula

[128] $$\int_{-\infty }^{\infty }\text{exp }[-\beta (e^{-\xi }+f\xi )]d\xi =\frac{1}{\zeta_{Q}(\beta /2)}\;{\left(\int_{-\infty }^{\infty }\text{exp }\left[-\frac{\beta }{2}(e^{-x}+fx)\right]\;dx\right)}^{2}$$

where (*ρ* = *βf*)

[129] $$\begin{array}{l} \zeta_{Q}(\beta /2)=\int_{-\infty }^{\infty }\frac{d\eta }{{\text{cosh}}^{\rho }(\eta /2)}\\ =2^{\rho }\frac{{\left[\Gamma (\rho /2)\right]}^{2}}{\Gamma (\rho )}. \end{array}$$

Here I have used a special notation *ζ_Q_*, which incidentally appears later again.

In [128] the phase integral at the reciprocal temperature *β* is related to the square of the integral at *β/*2, or rather to the integral for one half of the potential, $\frac{1}{2}(e^{-x}+fx)$,

[130] $$\begin{array}{l} Q(\beta /2)=\int_{-\infty }^{\infty }e^{-\frac{\beta }{2}(e^{-q}+f_{q})}dq\\ ={\left(\frac{\beta }{2}\right)}^{-\rho /2}\Gamma (\rho /2). \end{array}$$

From the above consideration, it seems clear that *ψ*(0) has something to do with *Q*(*β/*2) above, though they look quite different from each other.

## Final result for *ψ*(0)

Since *ψ*(0) is related to the phase shift *θ*(*p*) of binary collision, we turn to dynamics rather than sticking to the configurational integral *Q*(*β*).

For the binary system interacting with the exponential interaction, we have

[131] $$\frac{1}{2}\;{\left(\frac{dx}{dt}\right)}^{2}+e^{-x}=E.$$

Integrating we get

[132] $$\begin{array}{l} t=\int_{\;}^{x}\frac{dx}{\sqrt{2(E-e^{-x})}}\\ =\frac{1}{\sqrt{2E}}\;\text{log}\;\frac{\sqrt{E}+\sqrt{E-e^{-x}}}{\sqrt{E}-\sqrt{E-e^{-x}}}. \end{array}$$

This is equivalent to

[133] $$\begin{array}{l} x=2\;\text{log}\;\frac{\text{cosh}(\sqrt{E/2}t)}{\sqrt{E}},\\ p=\frac{dx}{dt}=\sqrt{2E}\;\text{tanh}(\sqrt{E/2}t). \end{array}$$

We now introduce the canonical transformation (*x,p*)→(*Q,P*) by setting $\sqrt{2E}=P,\;\sqrt{2E}t=Q$:

[134] $$\begin{array}{c} x=2\;\text{log}\;\frac{\text{cosh}(Q/2)}{P/\sqrt{2}},\;\;\;p=P\;\text{tanh}(Q/2),\\ \frac{\partial (x,p)}{\partial (Q,P)}=1,\;\;\;dx\;dp=dQ\;dP. \end{array}$$

We see

[135] $$\frac{1}{2}p^{2}+e^{-x}=\frac{1}{2}P^{2}$$

which indicates that *P* is the momentum responsible for the binary collision. The lattice Hamiltonian

[136] $$H=\frac{p^{2}}{2}+e^{-x}+fx$$

is then separated as

[137]    H =HP+HQ,HP =P22-2f log(P/2),HQ=2f log(Q/2).

Therefore we have the partition function *ζ*(*β*)

[138] $$\zeta (\beta )=\zeta_{P}(\beta )\zeta_{Q}(\beta ),\;\;\;\rho =\beta f,$$

with

[139] $$\begin{array}{l} \zeta_{P}(\beta )=\int_{0}^{\infty }dP\;e^{-\beta H_{P}}=\frac{1}{2\rho }\int_{0}^{\infty }e^{-\beta P^{2}/2}P^{2\rho }dP\\ =\sqrt{\frac{2\pi }{\beta }}\;\frac{\beta^{-\rho }}{2^{2\rho }}\frac{\Gamma (2\rho )}{\Gamma (\rho )},\\ \zeta_{Q}(\beta )=\int_{-\infty }^{\infty }dQe^{-\beta H_{Q}}=\int_{-\infty }^{\infty }\frac{dQ}{{\text{cosh}}^{2\rho }(Q/2)}\\ =2^{2\rho }\frac{{\left[\Gamma (\rho )\right]}^{2}}{\Gamma (2\rho )}. \end{array}$$

Changing *β* to *β/*2 (and *ρ* = *βf* to *ρ/*2), we see

[140] $$\begin{array}{l} \zeta_{P}(\beta /2)=\sqrt{\frac{\pi }{\beta }}\;\frac{\beta^{-\rho /2}}{2^{\rho /2-1}}\frac{\Gamma (\rho )}{\Gamma (\rho /2)},\\ \zeta_{Q}(\beta /2)=2^{\rho }\frac{{\left[\Gamma (\rho /2)\right]}^{2}}{\Gamma (\rho )}. \end{array}$$

Comparing [123] and [140], it seems quite natural to think that *e^ψ^*^(0)^ and 1*/ϕ*(0) are related to

[141] $$\zeta_{P}(\beta /2)=\sqrt{\frac{\pi }{\beta }}e^{\psi (0)},\;\;\;\zeta_{Q}(\beta /2)=\sqrt{\frac{8}{\pi }}\;\frac{1}{\varphi (0)}.$$

Among these, *ζ_P_* (*β/*2) is the phase integral for *H_P_* in [137], and according to [106], *ψ*(0) is the integral [121] over a function of the phase shift of the binary collision. Therefore the first equation of [141] must be explained along this line. The physical meaning of *ζ_Q_* is not yet clear.

## Concluding remark

If we replace *ψ*(0) and *ϕ*(0) in [120] by *ζ_P_* (*β/*2) and *ζ_Q_*(*β/*2) of [141], and *e*^−^*^βμ^* by the phase integral *ζ*(*β*), we have

[142] $$\zeta (\beta )=\sqrt{\frac{\beta }{8\pi }}\zeta_{Q}(\beta /2){\left[\zeta_{P}(\beta /2)\right]}^{2}$$

or, using [138], we get

[143] $$\zeta (\beta )=\sqrt{\frac{\beta }{8\pi }}\;\frac{1}{\zeta_{Q}(\beta /2)}{\left[\zeta (\beta /2)\right]}^{2}.$$

The last equation gives the partition function *ζ*(*β*) at the temperature *β*, as a function of the partition function $\zeta (\frac{\beta }{2})$ at $\frac{\beta }{2}$, provided if $\zeta_{Q}(\frac{\beta }{2})$ is known.

## Figures and Tables

**Fig. 4(a) f4a-pjab-80-445:**
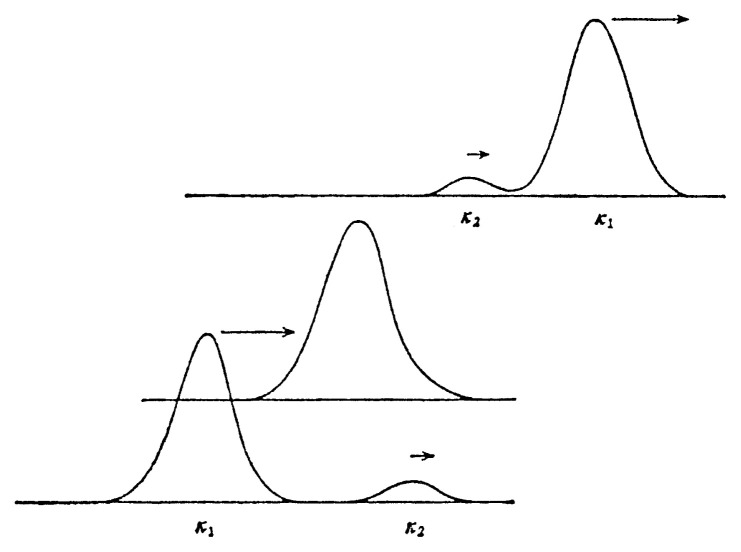
Overtaking collision when the difference in height is remarkable (lower solution is absorbed and sent out).

**Fig. 4(b) f4b-pjab-80-445:**
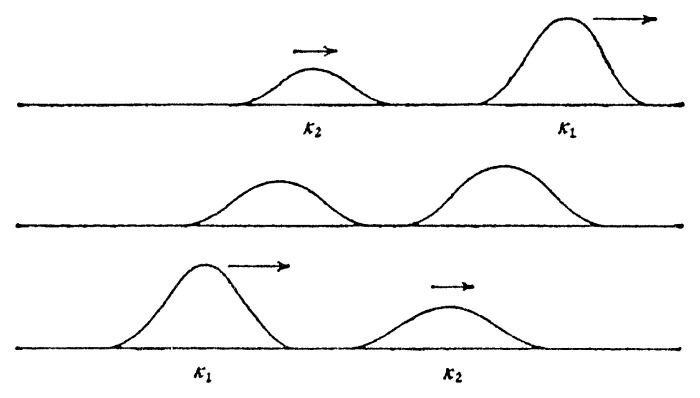
Overtaking collision when the difference in height is small (heigher soliton gets lower, while lower soliton gets higher and their roles are interchanged).
